# Beyond Bacteria: Bacteriophage-Eukaryotic Host Interactions Reveal Emerging Paradigms of Health and Disease

**DOI:** 10.3389/fmicb.2018.01394

**Published:** 2018-06-27

**Authors:** Anushila Chatterjee, Breck A. Duerkop

**Affiliations:** Department of Immunology and Microbiology, University of Colorado School of Medicine, Aurora, CO, United States

**Keywords:** bacteriophage, virome, host–microbe interactions, phage–bacteria interactions, microbiota, microbiome, phage immunity

## Abstract

For decades, a wealth of information has been acquired to define how host associated microbial communities contribute to health and disease. Within the human microbiota this has largely focused on bacteria, yet there is a myriad of viruses that occupy various tissue sites, the most abundant being bacteriophages that infect bacteria. Animal hosts are colonized with niche specific microbial communities where bacteria are continuously co-evolving with phages. Bacterial growth, metabolic activity, pathogenicity, antibiotic resistance, interspecies competition and evolution can all be influenced by phage infection and the beneficial nature of such interactions suggests that to an extent phages are tolerated by their hosts. With the understanding that phage-specific host–microbe interactions likely contribute to bacterial interactions with their mammalian hosts, phages and their communities may also impact aspects of mammalian health and disease that have gone unrecognized. Here, we review recent progress in understanding how bacteria acquire and tolerate phage in both pure culture and within complex communities. We apply these findings to discuss how intra-body phages interact with bacteria to influence their eukaryotic hosts through potential contributions to microbial homeostasis, mucosal immunity, immune tolerance and autoimmunity.

## Introduction

The human body hosts a complex and dynamic consortia of microbes consisting of bacteria, archaea, fungi, viruses and protozoa (Zou et al., 2016; Blum, 2017; Chabe et al., 2017; Huseyin et al., 2017; Koskinen et al., 2017; Raymann et al., 2017). Among the core members of the human microbiota, bacteria have garnered significant attention because of their contributions to human physiology and disease (Hooper and Gordon, 2001; Belkaid and Hand, 2014; Byrd et al., 2018; Zhao and Elson, 2018). The emergence of culture-independent approaches and techniques for viral enrichments from complex microbial samples has identified a vast consortium of understudied viruses within host associated microbiotas (Virgin, 2014).

The onset of the “omics” revolution led by 16s rDNA sequencing rapidly advanced our ability to survey the bacterial component of the microbiota in unprecedented detail. Extending from these studies, the implementation of metagenomic DNA sequencing revealed a robust viral component to the microbiota and identified bacteriophages (phages) as dominant members. In humans, phages populate most surfaces including skin (Foulongne et al., 2012; Oh et al., 2016), the oral cavity (Willner et al., 2011; Pride et al., 2012; Abeles et al., 2014), lungs (Willner et al., 2009; Dickson and Huffnagle, 2015), the intestine (Reyes et al., 2010; Minot et al., 2011; Manrique et al., 2016) and the urinary tract (Santiago-Rodriguez et al., 2015; Miller-Ensminger et al., 2018). Phage–bacteria interactions have been studied in varying detail *in vitro* (Chevallereau et al., 2016; Leskinen et al., 2016; Mojardin and Salas, 2016), however, little work to date has revealed insights into how phages interact with their bacterial hosts in human and animal systems. Body sites are endowed with unique characteristics including microenvironments that can define unique physiologies, thus it is conceivable that in some instances phage–bacteria interactions *in vivo* may be distinct from what has been studied in the laboratory.

Within the human body, phages infect bacterial hosts and undergo lytic replication and phage particle biogenesis to synthesize new infectious phages or integrate into the host bacterial genome as quiescent lysogenic prophages that are propagated vertically during cell division (Weinbauer, 2004; Hobbs and Abedon, 2016). Environmental cues such as nutrients, antibiotics and reactive oxygen species are well documented *in vitro* inducers of prophage excision from bacterial genomes, yet we know very little about the *in vivo* cues that promote prophage excision or those that influence the maintenance of lysogeny (DeMarini and Lawrence, 1992; Duerkop et al., 2012; Matos et al., 2013).

Considering the plethora of lytic and lysogenic phages that associate with humans, these phages are poised to have a significant impact on human physiology during both health and disease. In fact, research using animal models indicate that the intestinal microbiota promotes phage genome evolution allowing phages to infect naïve bacterial species and consequently fostering intra-body persistence (De Sordi et al., 2017). Hence, the cross-talk between resident bacteria and phages potentially contributes to the maintenance of microbial homeostasis within the human body. In this review, we will discuss phage–bacterial interactions within the context of host-associated microbial communities and will explore the underlying reasons for the evolution of phage tolerance in both bacteria and animals.

## Phage–Bacterial Collaboration: Benefits of Befriending the Enemy

Predatory lytic phages play crucial roles in maintaining diversity within microbial ecosystems (Maslov and Sneppen, 2017). Lytic phages adsorb to susceptible bacteria and subsequently infect and kill these bacteria. According to the classic “kill-the-winner” model, abundant bacterial species in a population have a greater possibility of encountering virulent phages and consequently face death, thus preventing niche monopoly by a single bacterial species (Thingstad, 2000; Rodriguez-Brito et al., 2010). Coculture studies using two competitive *Pseudomonas* strains demonstrated that phages enable the less competitive bacterial species to persist by infecting the more dominant species at a higher frequency, thus influencing community composition (Brockhurst et al., 2006). The contribution of lytic phages to bacterial diversity and richness in host associated environments is unknown and it is unclear whether bonafide “kill-the-winner” dynamics apply (Reyes et al., 2010; Allen et al., 2011; Abedon, 2012).

**FIGURE 1:**
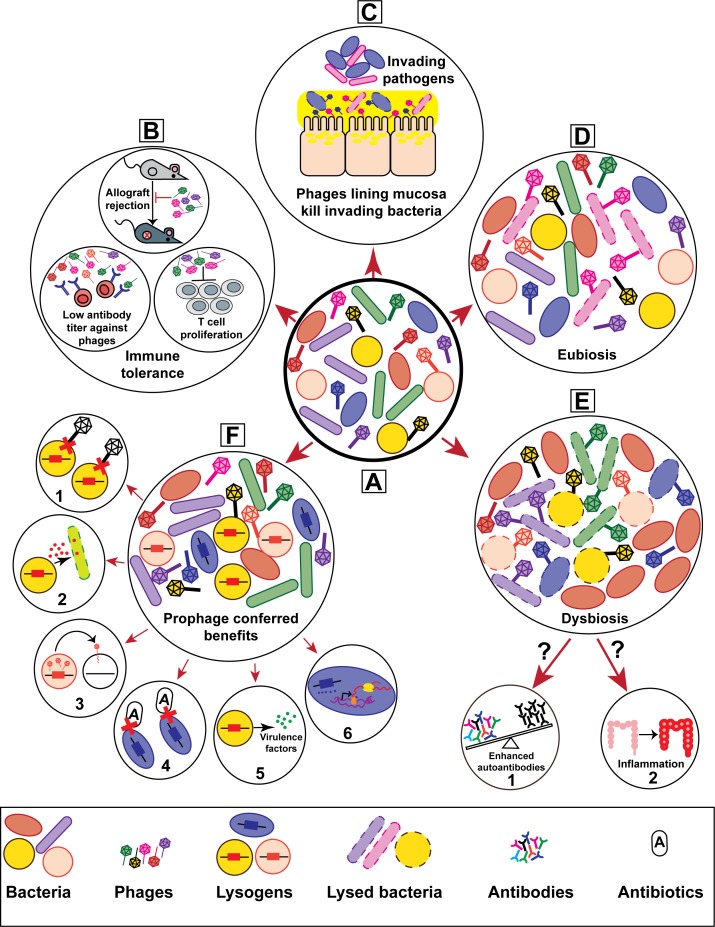
Bacteriophages contribute to the genetic and physiological traits of their hosts thereby influencing host–microbe interactions. **(A)** Schematic representation of a healthy mammalian microbiota consisting of heterogeneous phage and bacterial populations. The prolonged and ubiquitous presence of phages in mammalian microbiotas are hypothesized to have considerable effects on health and disease. Phage-driven impacts on mammalian hosts include **(B)** immune tolerance, **(C)** mucosal immunity, and **(D)** homeostatic eubiosis. Altered phage diversity and richness have been suggested to drive **(E)** bacterial dysbiosis, potentially leading to **(E-1)** autoimmune progression in type I diabetes and **(E-2)** inflammation during inflammatory bowel disease. On the other hand, phage infection endows bacteria with multiple features that alter bacterial interactions with their mammalian hosts. **(F)** Lysogenic conversion via the acquisition of prophages can increase bacterial host fitness. Prophage provided traits include **(F-1)** superinfection immunity, **(F-2)** elimination of bacterial competitors, **(F-3)** horizontal gene transfer, **(F-4)** enhanced antibiotic resistance, **(F-5)** virulence, and **(F-6)** altered gene expression.

Lysogenic phages integrated into host bacterial chromosomes can constitute up to 20% of bacterial genomes (Casjens, 2003), raising the question of why some bacteria tolerate such high burdens of phage DNA? Phage tolerance is likely supported by the potential positive outcomes bestowed upon the bacterium while harboring the viral DNA (**Figure 1**). Specifically, bacteria have co-evolved with their phages to benefit from the inclusion of viral genes within their genomes, which can aid in bacterial fitness, pathogenesis and adaptation to changing environments. Within this context, we will briefly discuss how bacteria benefit from their associated phages and we direct the readers to more recent comprehensive reviews on this subject (Roossinck, 2011; Obeng et al., 2016; Harrison and Brockhurst, 2017; Touchon et al., 2017).

Phages are vehicles for the horizontal transfer of genes which upon acquisition can influence individual and bacterial community phenotypes (reviewed in Touchon et al., 2017). For instance, prophages have been shown to confer pathogenic and antibiotic resistance traits for their bacterial hosts (Matos et al., 2013; Obeng et al., 2016; Lekunberri et al., 2017). Examples include a myriad of toxins which are encoded within prophage elements, the most well studied being the Shiga toxin-encoding prophages of *Escherichia coli* (STEC) which cause fatal gastrointestinal infections in humans (Shaikh and Tarr, 2003). Interestingly, a recent report showed that in addition to toxin production, the carriage of Shiga toxin-encoding prophages enhances antimicrobial tolerance of STEC by modifying the bacterium’s metabolism (Holt et al., 2017). Additionally, toxin encoding phages from numerous pathogenic bacteria have been demonstrated to transduce and lysogenize non-pathogenic bacteria, converting them to virulent strains (Faruque et al., 1999; Schmidt et al., 1999; Broudy and Fischetti, 2003).

The induction of lysogenic prophages from bacterial chromosomes has been linked to pathogen fitness and virulence. Production of EfCIV583, a satellite prophage who’s DNA is packaged into the capsid of the helper phage vB_EfaS_V583-P1 in *Enterococcus faecalis* strain V583, allows the host bacterium to compete with non-lysogenic peers (Duerkop et al., 2012; Matos et al., 2013). Another example from mixed culture experiments shows that prophage excision and subsequent phage mediated lysis of a subpopulation of host bacteria results in the timed release of bacteriocins that kill bacterial competitors and clear the niche for the phage-harboring bacteria (Nedialkova et al., 2016). Spontaneous prophage induction can also prime a shift in bacterial lifestyle from independently growing bacterial cells to organized cellular aggregates termed biofilms (reviewed in Nanda et al., 2015). The lungs of cystic fibrosis (CF) patients are colonized by sessile communities of *Pseudomonas aeruginosa* whose structural organization resemble biofilms (Bjarnsholt et al., 2013). Within these *P. aeruginosa* biofilms prophage induction occurs resulting in the release of the filamentous phage Pf4 (Secor et al., 2017). Pf4 phages promote biofilm assembly, facilitate persistence of the host bacteria in the lung and modulate inflammatory responses to promote chronic infections (Rice et al., 2009; Secor et al., 2015, 2017). Hence, prophage induction and subsequent phage-driven bacterial cell lysis provides a fitness benefit to the host bacterial community.

The integration of lysogenic phages into bacterial chromosomes can disrupt genes, thus altering phenotypes and in some cases altering bacterial fitness (Coleman et al., 1991; Bernhardt et al., 2000; Brussow et al., 2004). In a phenomenon termed reversible active lysogeny, prophage excision from the bacterial genome re-activates a host gene without activating the phages lytic cycle which promotes host adaptation (Feiner et al., 2015). For example, a *Listeria monocytogenes* prophage integrated within the master regulator of competence gene *comK*, during intracellular growth excises from the *L.* *monocytogenes* genome to restore the *comK* reading frame. The bacterium represses phage lysis and produces a functional ComK protein to promote immune evasion (Rabinovich et al., 2012). In the case of reversible active lysogeny, prophages provide gain-of-function phenotypes at the cost of the bacterium which must maintain the prophage element both within its dormant integrated form and repress its lytic functions after excision. Such costs are likely mitigated considering maintenance of these phages benefit the bacterium. The mechanisms driving these types of co-evolution are unclear, however, the acquisition and selection of phages within bacterial genomes shows that tolerance of phage lysogeny can promote context dependent attributes that promote bacterial adaptation to diverse environments.

Bacteria have evolved multiple defense strategies to restrict virulent phage predation and prophage acquisition (Labrie et al., 2010). Examples include restriction-modification (RM) and CRISPR (clustered regularly interspaced short palindromic repeats)/Cas (CRISPR-associated protein) systems that target invading phage DNA for destruction (Tock and Dryden, 2005; Barrangou et al., 2007; Sorek et al., 2008; Abedon, 2012). The absence of functional CRISPR-Cas has been linked to the emergence of multidrug resistant bacteria (Palmer and Gilmore, 2010; van Belkum et al., 2015), suggesting that the inability of bacteria to protect their genomes from foreign mobile elements including phages promotes bacterial adaptation to defined environmental conditions. Conversely, a recent study demonstrated that a conditional CRISPR/Cas system of *Staphylococcus epidermidis* evolved to favor prophage acquisition promoting a type of selective phage tolerance by degrading lytic phage DNA but allowing phage lysogeny (Goldberg et al., 2014) and RM systems have been shown to advocate prophage acquisition by postponing the onset of viral replication until bacterial population density has reached a point where the probability of lysogenic conversion is high (Pleska et al., 2018). These studies suggest that even in the presence of functional CRISPR-Cas and RM systems there may be preferences for specific DNAs that benefit bacteria in a context dependent manner.

## Bacteriophage-Eukaryote Interactions: the Tip of the Iceberg

Although metagenomic studies have revealed phages as one of the most abundant components of the human microbiota (Reyes et al., 2010; Minot et al., 2011; Foulongne et al., 2012; Pride et al., 2012; Oh et al., 2014; Dickson and Huffnagle, 2015; Santiago-Rodriguez et al., 2015; Oh et al., 2016), information on the interactions of phages with animal cells and how phages contribute to health and disease is limited. In this section, we will discuss recent literature related to how phages interact with eukaryotic hosts and how these interactions influence host immunity.

Over a decade ago, it was appreciated that intestinal phages breach the physical barrier of the mammalian intestine (Górski et al., 2006b). Compromised intestinal epithelial integrity during inflammation provides phages with access to the bloodstream and consequently their spread to different tissues (Handley et al., 2012). However, in the absence of intestinal barrier distress phages still migrate to host restricted sites such as peripheral blood and organs. It has been proposed that phages are naturally internalized into eukaryotic cells (Duerkop and Hooper, 2013; Tian et al., 2015; Zhang et al., 2017), however, the mechanisms behind phage uptake by eukaryotic cells are just beginning to be explored. A long held theory behind phage internalization by eukaryotic cells is that these events were preceded by the entry of phage-infected bacterial cells that transport phages (Hsia et al., 2000; Johnston, 2002; Duerkop and Hooper, 2013). Recently phages have been shown to gain access to epithelial cells directly through active transcytosis in the absence of their bacterial host (Nguyen et al., 2017). Phages were shown to permeate the apical surface of epithelial cells via endocytosis, become compartmentalized and finally exocytosed through the basal side of the cell (Nguyen et al., 2017). In a separate study, *Escherichia coli* phage PK1A2 was shown to recognize and bind neuroblastoma cells displaying polysialic acids on their cell surface (Lehti et al., 2017). Following adhesion, phage PK1A2 is internalized by the endolysosomal pathway and are eventually degraded in the lysosome (Lehti et al., 2017). It is hypothesized that during these internalization events, phages may escape lysosomal destruction and potentially create opportunities for trans-kingdom genetic exchange or stimulate cellular immunity (Duerkop and Hooper, 2013; Lehti et al., 2017). In another example of potential *trans*-kingdom interactions, it has been proposed that phages act as an additional layer of non-host derived immunity against incoming pathogens at mucosal surfaces by binding to mucin glycoproteins [(Barr et al., 2013a) and discussed later]. Carbohydrate modifications, including sialic acids and various glycosylations are abundant in host derived mucins (Royle et al., 2008). Determining if phage adhesion to the sugar epitopes of host mucins is a common strategy by which phages interact with eukaryotic mucosal surfaces should reveal possible mechanisms behind the *in vivo* translocation and dispersal of phages within the human body.

As phages are significant reservoirs of genetic diversity and considering phages are capable of entering eukaryotic cells, this raises questions about the possibility of bidirectional trans-kingdom gene exchange between phages and their animal hosts. Multicellular eukaryotes have been reported to harbor phage capsid gene orthologs in their genomes that resemble phages of the obligate intracellular pathogen *Chlamydophila pneumoniae* (Rosenwald et al., 2014). Phages have also been implicated in the dissemination of bacterial aerolysin and lysozyme genes within eukaryotic hosts (Moran et al., 2012; Metcalf et al., 2014). Conversely, metazoan-like gene modules whose functions have yet to be defined have been found in phages of the insect parasite *Wolbachia* (Bordenstein and Bordenstein, 2016). Together, these observations suggest the potential for the genetic interplay between phages and eukaryotes that contribute to trans-kingdom evolution (**Figure 1**).

Commensal bacteria regulate various facets of host immunity, yet there are significant gaps in our understanding of the mechanisms driving microbiota mediated immune regulation (Mazmanian et al., 2005; Ivanov et al., 2008; Arpaia et al., 2013; Fung et al., 2017; Novince et al., 2017; Schnupf et al., 2017). Considering phages influence the assembly of microbial communities and modulate bacterial diversity in various ecosystems (Barr et al., 2013b; Koskella and Meaden, 2013), perhaps phage–bacteria interactions can direct the host immune response. Phages could potentially modulate immune interactions between a eukaryotic host and its microbiota by providing novel traits within subpopulations of bacteria or by causing shifts in the resident bacterial community composition through targeted killing of defined community members (**Figure 1**). Given the prevalence of phages at multiple tissue sites within the human body, it is likely that phages play an unrecognized role in promoting the development and activity of the immune system through interactions with their host bacteria. It is possible that lytic phages could directly stimulate antiviral innate immunity by engaging nucleic acid sensors or inadvertently by killing their bacterial hosts and releasing soluble bacterial antigens that stimulate pattern recognition receptors. If either of these scenarios were true, this would have profound implications for the development of lytic phages as antibacterial therapeutics.

Changes in phage community composition occur during human disease. For example, the diversity and composition of intestinal phages is significantly different between healthy individuals and patients with inflammatory bowel diseases (IBD) (Wagner et al., 2013; Norman et al., 2015). Individuals with IBD have reduced enteric bacterial diversity relative to the healthy individuals. However, alterations in bacterial richness does not always correlate with the dramatic phage expansion associated with IBD (Norman et al., 2015), suggesting signals from the immune system may directly influence phage abundances. Additionally, alterations in enteric phage populations was observed prior to the development of autoantibodies in children who were predisposed to develop Type I diabetes (Zhao et al., 2017). These disease-related shifts in phage community composition suggest a potential role for intestinal phages in the development of bacterial dysbiosis. Although phages have been implicated in diseases associated with bacterial dysbiosis, it is unclear if phages directly contribute to inflammation and autoimmune disease by altering microbial homeostasis (**Figure 1**).

The collaboration between phages and their animal hosts to eliminate deleterious bacteria is opening new avenues for the study of host–microbe interactions during health and disease. In a recent study, researchers revealed a neutrophil-phage alliance that together cleared multi-drug resistant *P. aeruginosa* in a lung infection mouse model (Roach et al., 2017). This study suggests that host innate immunity may be more effective at clearing pathogenic bacteria with help from lytic phages. *In vitro* tissue culture studies suggest that phages protect epithelial mucosal surfaces from invading bacteria (Barr et al., 2013a, 2015). These studies propose that phages with binding affinity for host mucins form a protective antibacterial defense called BAM (bacteriophage adhesion to mucus) which serves as a non-host derived innate immunity at mucosal surfaces. According to the BAM model, adhesion of phages to mucin glycoproteins and subsequent subdiffusive movement through the mucus layer concentrates phages at mucosal surfaces. An enrichment of phages in the mucosa may provide protection against bacterial invaders and limit pathogen colonization (Barr et al., 2013a, 2015). Although, these findings suggest that phages contribute to host defenses (**Figure 1**), their function in promoting mucosal health remain to be explored.

An increasing body of data suggests that phages engage in interactions with mammalian immune cells and modulate different aspects of host immune responses. Phages are weakly immunogenic and the adaptive immune system produces low titers of phage-neutralizing antibodies without mounting an inflammatory response (Dabrowska et al., 2006; Górski et al., 2012; An et al., 2014; Majewska et al., 2015). Knowing that phages are ubiquitous within host associated microbiotas and possibly within the host systemic environment, it is possible that immune tolerance to phages occurs due to the continued exposure of the immune system to phages. Several studies propose a role for phages in promoting immune tolerance by downregulating T cell proliferation, through the reduction of antibody production and in the prevention of allogenic transplant rejection in animal models (Górski et al., 2006a,b, 2007; McVay et al., 2007). For a comprehensive discussion on the effect of phages on immune-modulation readers are directed to a recent review by Górski et al. (2017).

## Concluding Remarks

Phages endow their host bacteria with competitive traits, facilitate adaptation for the colonization of new niches and promote bacterial evolution. It is becoming increasingly clear that the impact of phages extend beyond their bacterial hosts and their potential influences on human health are just beginning to be explored (De Paepe et al., 2014; Norman et al., 2015; Manrique et al., 2016; Wahida et al., 2016). Recent studies bring to light concepts for how bacteria and animals have co-evolved to tolerate phages through beneficial interactions that may dictate the outcomes of host–microbe associations. Through these interactions phages and their communities hold substantial promise as modulators of human health and disease.

Moving forward, studies that employ modern “omics” technologies to study the microbiota such as metagenomics, transcriptomics and proteomics should by default incorporate analyses of phage communities. Alongside such studies, researchers must make efforts through the use of mouse models and *in vivo* defined microbial communities to move beyond descriptive studies and begin providing mechanistic details into how phages interact with host-associated bacteria and how these interactions influence immunity and physiology. Furthermore, specific attention should be given to the mammalian host responses that drive the assembly of phage community composition within the microbiota and how these signals influence phage interactions with their bacterial hosts. Only after these basic questions are explored can we begin to understand how to harness phages for the manipulation of bacterial communities that promote human health.

## Author Contributions

All authors listed have made a substantial, direct and intellectual contribution to the work, and approved it for publication.

## Conflict of Interest Statement

The authors declare that the research was conducted in the absence of any commercial or financial relationships that could be construed as a potential conflict of interest.
